# Crystal structures of the hexa­fluorido­phosphate salts of the isomeric 2-, 3- and 4-cyano-1-methyl­pyridinium cations and determination of solid-state inter­action energies

**DOI:** 10.1107/S2056989018011003

**Published:** 2018-08-24

**Authors:** Joel T. Mague, Erin Larrabee, David Olivier, Francesca Vaccaro, Kevin E. Riley, Lynn V. Koplitz

**Affiliations:** a Department of Chemistry, Tulane University, New Orleans, LA 70118, USA; b Department of Chemistry, Loyola University, New Orleans, LA 70118, USA; c Department of Chemistry, Xavier University of Louisiana, New Orleans, LA 70125, USA

**Keywords:** crystal structure, hexa­fluorido­phosphate, cyano­pyridinium salts, hydrogen bonds, DFT calculations

## Abstract

The synthesis and crystal structures of the isomeric mol­ecular salts 2-, 3- and 4-cyano-1-methyl­pyridinium hexa­fluorido­phosphate, C_7_H_7_N_2_^+^·PF_6_^−^, are reported. In 2-cyano-1-methyl­pyridinium hexa­fluorido­phosphate, C—H⋯F hydrogen bonds form chains extending along the *c*-axis direction, which are associated through C—H⋯F hydrogen bonds and P—F⋯π(ring) inter­actions into stepped layers. For 3-cyano-1-methyl­pyridinium hexa­fluorido­phosphate, corrugated sheets parallel to [001] are generated by C—H⋯F hydrogen bonds and P—F⋯π(ring) inter­actions. The sheets are weakly associated by a weak inter­action of the cyano group with the six-membered ring of the cation. In 4-cyano-1-methyl­pyridinium hexa­fluorido­phosphate, C—H⋯F hydrogen bonds form a more open three-dimensional network in which stacks of cations and of anions are aligned with the *b*-axis direction. Dispersion-corrected density functional theory (DFT-D) calculations were carried out in order to elucidate some of the energetic aspects of the solid-state structures. The results indicate that the distribution of charge within a mol­ecular ionic cation can play a large role in determining the strength of a cation–anion inter­action within a crystal structure. Crystals of 2-cyano-1-methyl­pyridinium hexa­fluorido­phosphate are twinned by a 180° rotation about the c* axis. The anion in 3-cyano-1-methyl­pyridinium hexa­fluorido­phosphate is rotationally disordered by 38.2 (1)° in an 0.848 (3):0.152 (3) ratio.

## Abstract 2

Metathesis of 2-, 3- and 4-cyano-1-methyl­pyridinium iodide with KPF_6_ in water generated the corresponding hexa­fluorido­phosphate salts, C_7_H_7_N_2_^+^·PF_6_^−^, whose crystal structures were determined. They feature a variety of weak inter­actions (C—H⋯F hydrogen bonds and P—F⋯π inter­actions). Dispersion-corrected density functional theory (DFT-D) calculations were carried out in order to elucidate some of the energetic aspects of the solid-state structures.

## Chemical context   

Our inter­est in the structural features of salts of the cyano-1-methyl­pyridinium cations (CMP) was generated by the significantly different melting behaviors of 3-CMP chloride and iodide (Koplitz *et al.*, 2003 ▸). This was attributed to a greater amount of C—H⋯N and C—H⋯*X* (*X* = Cl^−^, I^−^) hydrogen bonding in the former, in part because all ions lie on mirror planess in the chloride salt while the cation planes are not parallel in the iodide. As a result, it was estimated that the stabilization is at least 1.9 kcal mol^−1^ more in the chloride than in the iodide. At that time, relatively few crystal structures of CMP salts had been published so in order to investigate the packing and non-covalent inter­actions for these cations in the solid state, structures of salts of the 2-, 3- and 4-CMP^+^ cations with a variety of anions including Br^−^ (Kammer *et al.*, 2012*b* ▸; Mague *et al.*, 2005 ▸; Nguyen *et al.*, 2015*b* ▸), I_3_^−^ (Nguyen *et al.*, 2016 ▸), I^−^ (Kammer *et al.*, 2012*a* ▸, 2013 ▸), ClO_4_^−^ (Nguyen *et al.*, 2014 ▸; Nguyen *et al.*, 2015*a* ▸; McCormick *et al.*, 2014 ▸), NO_3_^−^ (McCormick *et al.*, 2013 ▸; Koplitz *et al.*, 2012 ▸) and BF_4_^−^ (Vaccaro *et al.*, 2015 ▸) were determined. In addition to structures with parallel sheets as for 3-CMP chloride, ones with inter­pentrating layers, wrinkled sheets and three-dimensional networks are found. We report here on the hexa­fluorido­phosphate salts of all three cations. More broadly, a better understanding of the manifestations of non-covalent inter­actions in crystalline organic salts will lead to improved predictions for useful substances in a variety of fields, including materials engineering and targeted drug design. Mapping the crystal structure space for heterocyclic cations in a variety of salts is a very important early step in this overall context.
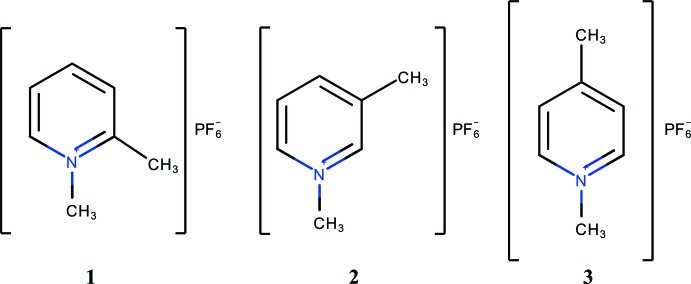


## Structural commentary   

**Figure 1:**
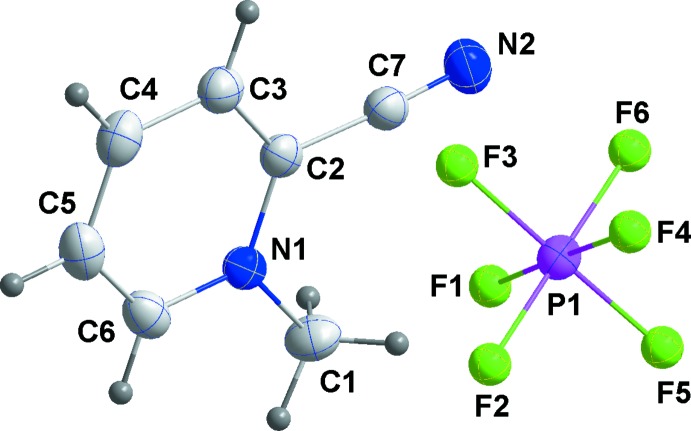
Perspective view of **1** with labeling scheme and 50% probability ellipsoids.

The mol­ecular structures of **1**–**3** [Chem scheme1] are unexceptional in that all three feature essentially planar cations and octa­hedral anions (Figs. 1 ▸, 2 ▸ and 3 ▸, respectively). The inter­est lies in their differing solid-state structures and inter­ionic inter­actions. First, **1** crystallizes in the centrosymmetric space group *P*2_1_/*n* while **2** and **3** are in the non-centrosymmetric space group *P*2_1_2_1_2_1_. Second, the number of inter­ionic inter­actions per asymmetric unit is six in **1**, five in **2** and four in **3**. With no mirror planes present, layer structures are not possible and the cation planes are canted with respect to [100] by ±63.19 (9)° in **1**, ±62.29 (8)° in **2** and ±31.41 (8)° in **3**. In **2** there is a close approach of the cyano group to the six-membered ring of the cation at *x* − 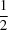, −*y* + 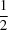, −*z* + 1 with an N2⋯centroid distance of 3.322 (4) Å and a C7—N2⋯centroid angle of 114.4 (3)°.

## Supra­molecular features   

**Figure 4:**
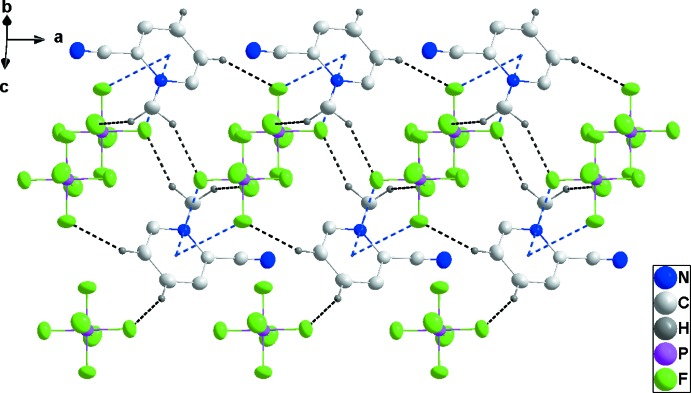
Side view of two cation and anion columns in **1** projected onto (021). C—H⋯F hydrogen bonds are shown as black dashed lines and P—F⋯π(ring) inter­actions by blue dashed lines.

**Figure 5:**
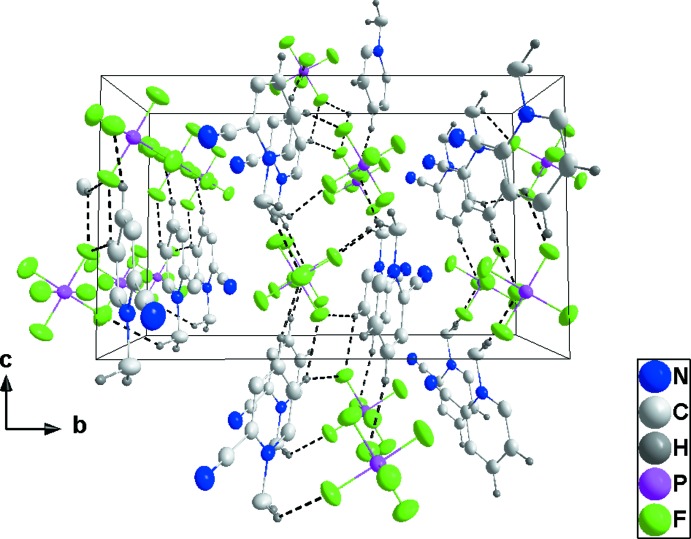
Packing of **1** viewed along the *a*-axis direction with C—H⋯F hydrogen bonds shown as dashed lines.

In **1**, one cation and one anion are associated through C4—H4⋯F6 and C5—H5⋯F5 hydrogen bonds (Table 1 ▸) and these units are linked by C1—H1*B*⋯F6 hydrogen bonds, forming chains extending along the *c*-axis direction. Pairs of chains are joined by C1—H1*A*⋯F4 hydrogen bonds and inter­actions of F5 and F6 with the six-membered rings at −*x* + 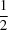, *y* − 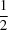, −*z* + 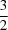 [F5⋯centroid = 3.4794 (17) Å, P1—F5⋯centroid = 105.65 (6)°, F6⋯centroid = 3.3569 (19) Å, P1—F6⋯centroid = 110.59 (8)°] of the cations (Table 1 ▸ and Fig. 4 ▸). The resulting double chains are further joined into stepped layers by C5—H5⋯F5 hydrogen bonds (Fig. 5 ▸).

**Figure 6:**
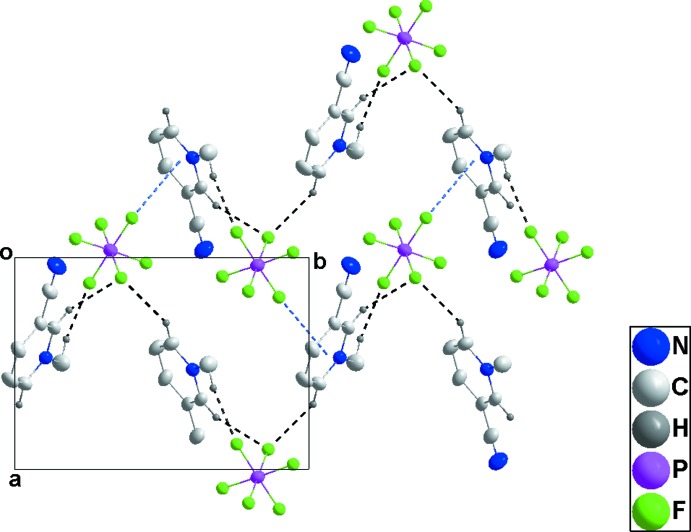
View of two adjacent cation–anion chains in **2** along the *c*-axis direction with C—H⋯F hydrogen bonds shown by black dashed lines.

**Figure 7:**
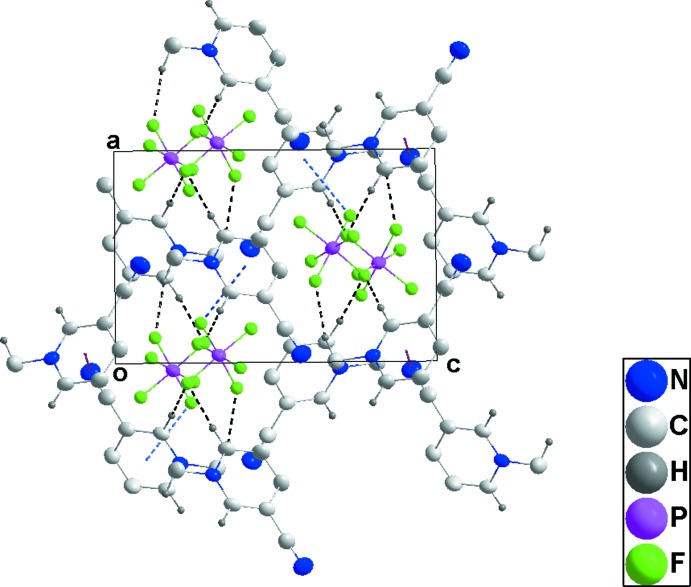
Packing of **2** viewed along the *b*-axis direction. C—H⋯F hydrogen bonds and P—F⋯π(ring) and C≡N⋯π(ring) inter­actions are shown, respectively, by black, blue and purple dashed lines.

For **2**, C1—H1*B*⋯F4, C2—H2⋯F6 and C6—H6⋯F6 hydrogen bonds (Table 2 ▸) form zigzag chains (Fig. 6 ▸), which are joined by the close inter­action of F1 with the six-membered rings of the cations [F1⋯centroid = 3.186 (3) Å, P1—F1⋯centroid = 123.67 (12)°, forming corrugated sheets parallel to [001]. These sheets are associated through the weak inter­action of the cyano group with the six-membered ring of the cation mentioned in the preceding section (Fig. 7 ▸).

**Figure 8:**
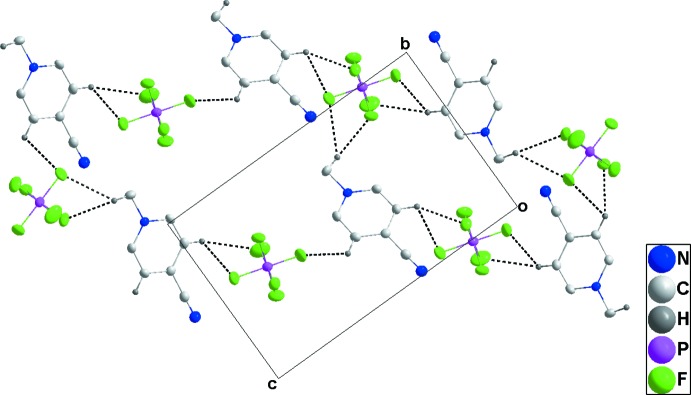
View of two adjacent cation–anion chains in **3** along the *a*-axis direction with C—H⋯F hydrogen bonds shown by black dashed lines.

In **3**, a relatively open, three-dimensional network structure in which stacks of cations and of anions are aligned with the *b*-axis direction is generated by C1—H1*C*⋯F1, C3—H3⋯F3 and C5—H5⋯F5 hydrogen bonds (Table 3 ▸ and Figs. 8 ▸ and 9 ▸).

## DFT studies   

Dispersion-corrected density functional theory (DFT-D) calculations were carried out in order to elucidate some of the energetic aspects of the CMP-PF_6_ structures. Calculations were carried out at the ωB97X-D/def2-TZVP level of theory (Jurečka *et al.*, 2007 ▸; Chai & Head-Gordon, 2008 ▸; Grimme, 2006 ▸; Schröder *et al.*, 2017 ▸). Here, all computations are carried out using the SMD (solvation model based on density) model in order to approximate the effect of the crystal environment (Marenich *et al.*, 2009 ▸). The dielectric constant of the CMP-PF_6_ crystals is currently unknown, so a dielectric constant of 4.0 was chosen as a generic value (as has been done in previous studies; Nguyen *et al.*, 2016 ▸). Although the inter­actions under consideration are between mol­ecular cations and anions, and complex stabilization is therefore attributable mainly to electrostatic forces, it is important that all attractive and repulsive forces (induction, dispersion, exchange) be modeled as well as possible. As DFT is known to describe dispersion inter­actions very poorly, here we have used a model incorporating an empirical dispersion term (-D2) in order to account for this shortcoming (Grimme, 2006 ▸). Dispersion plays a substantial role in stabilizing all non-covalent complexes (Riley *et al.*, 2010 ▸; Johnson *et al.*, 2010 ▸) and is known to be especially important in larger aliphatic and aromatic mol­ecules (Sedlak *et al.*, 2013 ▸). It has been shown that the parameterizations of empirical dispersion terms, which are generally established from gas-phase benchmark data, remain essentially unchanged when implicit solvent models, such as SMD, are used (Riley *et al.*, 2007 ▸).

**Figure 10:**
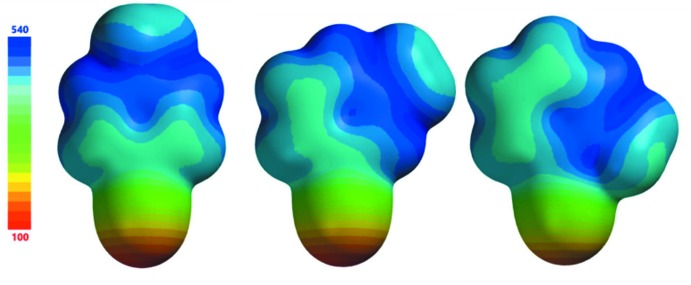
Electrostatic potential maps (kcal mol^−1^) for the 4-CMP^+^ (left), 3-CMP^+^ (center) and 2-CMP^+^ (right) cations. Note the large range of 440 kcal mol^−1^. The strong electron-withdrawing ability of the cyano group results in a significantly less positive partial charge for that part of the mol­ecular ion.

**Figure 11:**
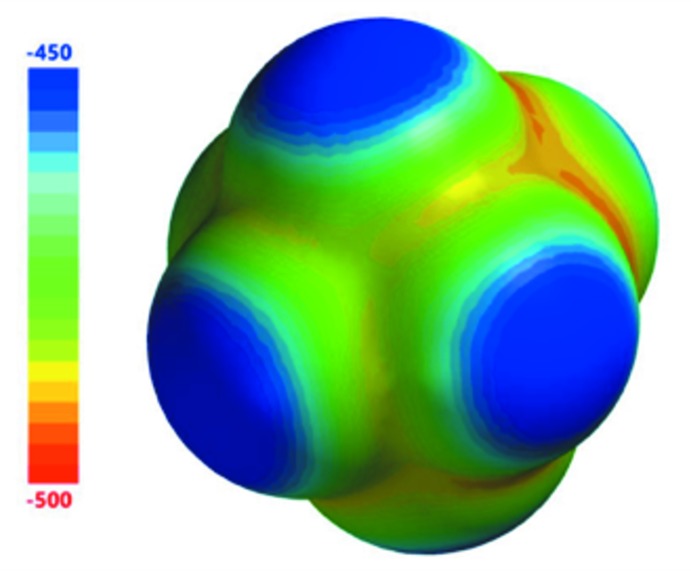
Electrostatic potential map (kcal mol^−1^) for the hexa­fluorido­phosphate anion. Note the relatively small range of 50 kcal mol^−1^.

Electrostatic potentials for the three CMP mol­ecular cations (Fig. 10 ▸) and the PF_6_^−^ anion (Fig. 11 ▸) were obtained at the B3LYP/6-311+G** level of theory. It has been shown that the quality of an electrostatic potential does not strongly depend on the level of theory (DFT or HF) or on the particular basis set used, so long as the basis set is sufficiently large (at least 6-31G*; Riley *et al.*, 2016 ▸). The most inter­esting aspect of these electrostatic potentials concerns the mol­ecular cations, for which there are seen to be large shifts in charge density from one part of the mol­ecular ion to another, with the most positive regions having potential values of 140 (**1**), 109 (**2**), and 108 (**3**) kcal mol^−1^ and the least positive regions having values of 529 (**1**), 533 (**2**), and 531 (**3**) kcal mol^−1^. This large shift in charge from one region to another is principally attributable to the high electron-withdrawing capacity of the cyano group, resulting in a less positive partial charge in that region of the mol­ecular ion. For all three mol­ecular cations, the most positively charged regions are those neighboring the CMP methyl groups (*i.e*. the H atoms that are *ortho*- to the methyl groups), with the exception of the region located between the methyl and cyano groups in **1**. As will be discussed below, the anisotropic distribution of charge throughout these mol­ecular cations has significant effects on the strengths of the inter­actions (Table 4 ▸) between these moieties and the PF_6_^−^ anions.

**Figure 12:**
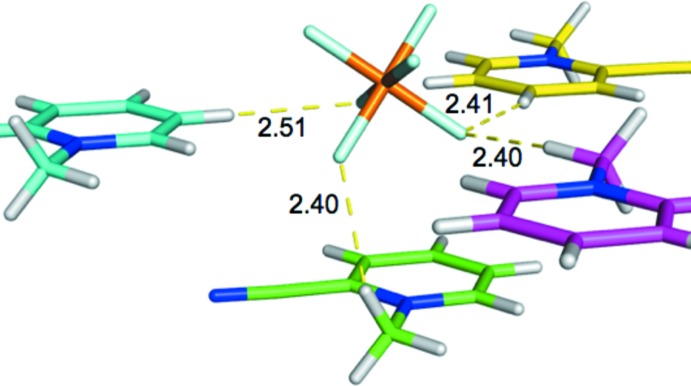
2-CMP^+^⋯PF_6_^−^ inter­actions. BLYP-D3/def2-TZVP/SMD inter­action energies (kcal mol^−1^) for these complexes are: −19.0 (green), −16.9 (cyan), −15.9 (purple), and −15.7 (yellow).

The shortest cation–anion contacts within the crystal structure of **1** are shown in Fig. 12 ▸. Here it is seen that three of the mol­ecular cations (shown in cyan, pink, and yellow) have aromatic rings that are coplanar with each other and are quasi-coplanar with three fluorine atoms from the PF_6_^−^ anion. In each case, two contacts are made between a cation H atom and one of the quasi-coplanar PF_6_^−^ fluorine atoms, although it should be noted that the longest contact in the inter­action involving the pink cation (3.59 Å) is substanti­ally longer than all other contacts (2.40–2.62 Å). Two of the shorter contacts involving aromatic hydrogen atoms (cyan, yellow) and one involving a methyl hydrogen atom (purple). The fourth close contact (green) is a stacking inter­action involves a 2-CMP cation located in a plane below PF_6_^−^ (as depicted), with a short C—H⋯F contact occurring between a methyl H atom and an anion F atom.

Unsurprisingly, among the four cation–anion pairs given in Fig. 12 ▸, the stacking contact (green) represents the strongest inter­action, with a binding energy of −19.0 kcal mol^−1^. The strength of this inter­action is mainly due to the large area of contact between cation and ion, with three F atoms within a distance of 3.4 Å from the cation. Without knowledge of the electronic density distribution, as reflected in the electrostatic potential, it might be assumed that the strongest inter­action among the PF_6_^−^ contacts with the three coplanar mol­ecular cations would be that involving the yellow cation, which exhibits the shortest contact distances with the PF_6_^−^ anion. Thus, it is somewhat surprising that this inter­action is actually predicted to be the *weakest* among the coplanar inter­actions, with an inter­action energy of −15.7 kcal mol^−1^. Surprisingly, even the coplanar inter­action with only one short H^+^⋯F^−^ contact (purple) exhibits slightly stronger attraction (−15.9 kcal mol^−1^), while the strongest inter­action (−16.9 kcal mol^−1^) occurs for the cyan cation, whose contact distances are slightly longer than those of the inter­action involving the yellow cation.

The counter-intuitive results described above can be explained by considering the distribution of charge on 2-CMP^+^, as reflected in the electrostatic potential. The most positive region of the 2-CMP^+^ cation encompasses the hydrogen neighboring the methyl group and the N—CH_3_ bond. Each of the two stronger complexes (cyan, purple) includes a contact between this strongly positive region of the electrostatic potential and a negative F atom. Conversely the shortest contact in the weaker of these complexes (yellow) involves the H atom that is *para*- to the methyl group, the least positively charged of the aromatic hydrogen atoms.

**Figure 13:**
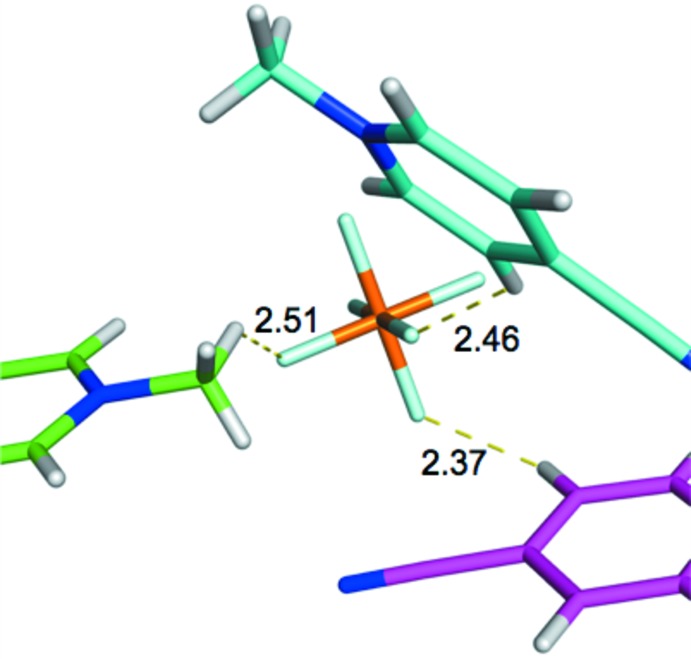
4-CMP^+^⋯PF_6_^−^ inter­actions. BLYP-D3/def2-TZVP/SMD inter­action energies (kcal mol^−1^) for these complexes are: −16.7 (green), −15.3 (cyan), −14.2 (purple).

The details of cation charge distribution are again seen to be important in determining inter­action strengths within the crystal structure of **3**. In Fig. 13 ▸ it is seen that the strongest inter­action involves the green 4-CMP^+^ mol­ecular cation (−16.7 kcal mol^−1^), whose shortest H^+^⋯F^−^ contact (involv­ing a methyl H atom) is the longest (2.51 Å) among the three inter­actions considered here. The enhanced strength of this inter­action, relative to the other two contacts, can be explained by the orientation of the 4-CMP^+^ cation relative to the PF_6_^−^ anion. As seen in Fig. 10 ▸, the regions neighboring the methyl group on the 4-CMP^+^ cation are significantly more positive than other regions of the mol­ecular ion. It is this highly positive region that forms contact with the PF_6_^−^ anion, as shown in Fig. 13 ▸. The weakest inter­action here involves the pink 4-CMP^+^ cation (−14.2 kcal mol^−1^), whose closest H^+^⋯F^−^ distance (2.37 Å) is the shortest among all contacts considered here. This contact involves a hydrogen atom that neighbors the 4-CMP cyano group, which is located in a region whose positive charge is relatively low.

**Figure 14:**
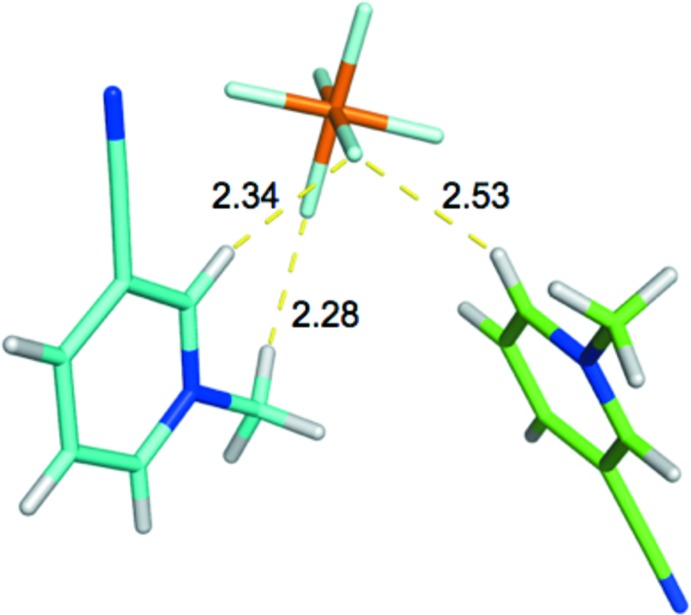
3-CMP^+^⋯PF_6_^−^ inter­actions. BLYP-D3/def2-TZVP/SMD inter­action energies (kcal mol^−1^) −17.8 for these complexes are: (green) and −16.6 (cyan).

The ordering of the inter­action strengths for the two complexes involving the 3-CMP^+^ cations, shown in Fig. 14 ▸, are also counter-intuitive. The inter­action with the shorter H^+^⋯F^−^ distances (cyan) represents the weaker of the two inter­actions. The stronger of the two inter­actions (green) involves the aromatic H atom that is *para*- to the cyano group, located on the most positive region of the cation. The proximity of this positive region to the anion is likely responsible for the stronger binding of this cation.

Results presented here indicate that the distribution of charge within a mol­ecular ionic cation can play a large role in determining the strength of a cation–anion inter­action within a crystal structure. It is presumed that careful inspection of electrostatic potentials becomes more important as the size of a cation increases and as strong electron-withdrawing groups, such as cyano groups, are introduced. Although not investigated here, similar trends are likely observed for larger mol­ecular anions.

## Database survey   

In addition to those compounds cited in the *Chemical context* section, there are 14 other structures in the CSD (Version 5.39; Groom *et al.*, 2016 ▸) containing cyano-1-methyl pyri­dinium cations. Of these, ten contain the 4-CMP cation and the other four the 3-CMP cation. Both 3- and 4-CMP[N(SO_2_CF_3_)_2_] are described with the former having a layer structure formed from cation chains involving C—H⋯N inter­actions between a ring hydrogen atom and the cyano group, which are bound to anion chains by C_ring_—H⋯O and C_meth­yl_—H⋯N hydrogen bonds. The layers have the tri­fluoro­methyl groups protruding from one face and the *para* ring hydrogens from the other. The latter has a three-dimensional network structure in which only the ring hydrogen atoms form C—H⋯O hydrogen bonds, leading to channels along the *a*-axis direction with the cyano, methyl and tri­fluoro­methyl groups forming the inner edges (Hardacre *et al.*, 2008 ▸). The co-crystal of 4-CMP[N(SO_2_CF_3_)_2_] with 1-methyl­napthalene has corrugated layers of alternating cations and anions with trifluromethyl groups protruding from both faces inter­spersed with layers of 1-methyl­napthalene (Hardacre *et al.*, 2010 ▸). In 4-CMP[CH_3_OSO_3_], C—H⋯O hydrogen bonds involving both aromatic and aliphatic H atoms form cation–anion chains along the *c*-axis direction, which are joined into double layers having the anion methyl groups protruding from both faces by C_meth­yl_—H⋯O hydrogen bonds (Hardacre *et al.*, 2008 ▸). A different structure is found in 4-CMP[Co(CO)_4_] where pairwise C_ring_—H⋯N inter­actions form dimers that are expanded into cross-linked zigzag chains by C_ring_—H⋯O hydrogen bonds with the anions (Bockman & Kochi, 1989 ▸). Cross-linked, zigzag chains are also found in 4-CMP[ZnI_4_], but here the chains are only cations and are formed by C_meth­yl_—H⋯N inter­actions. The anions serve to cross-link them through C_ring_—H⋯I and C_meth­yl_—H⋯I inter­actions (Glavcheva *et al.*, 2004 ▸). Another example of a layer structure is in [4-CMP]_2_{Cu[S_2_C_2_(CN)_2_]_2_} where alternating cation–anion chains are formed with half of the cations and the anions through C_ring_—H⋯N hydrogen bonds. The remaining cations use C_ring_—H⋯N hydrogen bonds to both cations and anions in the chains to form a three-dimensional network (Wang *et al.*, 2012 ▸).

The remaining structures feature large anions, but this does not necessarily isolate the cations from each other. In 4-CMP[{HB(3,5-di­methyl­pyrazol­yl)_3_}Mo(CO)_3_], the cations form dimers as in 4-CMP[Co(CO)_4_] and are associated with the anions through C_ring_—H⋯O hydrogen bonds as well as a π–π stacking inter­action with one of the pyrazolyl rings (Bockman & Kochi, 1992 ▸). An entirely different structure is seen in {(4-CMP)_2_[Cu_4_(μ_3_-I)(μ-I)_2_]}_*n*_ where zigzag chains of cations formed by C_ring_—H⋯N hydrogen bonds are arranged at right angles to one another between chains of anions and link the latter through C_meth­yl_—H⋯I inter­actions (Chan *et al.*, 2012 ▸). Similar zigzag chains of cations are found in {(3-CMP)[Ag_4_(μ_4_-I)_2_(μ-I)_2_(μ-I)]}_*n*_ but here they are all coplanar in a layer structure where cation and anion layers alternate (Yu *et al.*, 2014 ▸). Details of the inter­ionic inter­actions in {(4-CMP)[Ag_2_I_3_]}_*n*_ (Shen *et al.*, 2014 ▸) and (3-CMP)BPh_4_ (Zhu & Kochi, 1999 ▸) are obscured by considerable disorder.

## Synthesis and crystallization   

**2-Cyano-1-methyl­pyridinium hexa­fluorido­phosphate (1)**

To a solution of 2.499 g (1.016 mmol) of 2-cyano-1-methyl pyridinium iodide (Kammer *et al.*, 2013 ▸) dissolved in 20 ml of deionized water was added 1.87 g (1.221 mmol) of solid potassium hexa­fluorido­phosphate with stirring. The white solid that precipitated was washed with a small qu­antity of ice-cold, deionized water and recrystallized from deionized water by slow evaporation under a gentle stream of nitro­gen. M.p. 379 K.

**3-Cyano-1-methyl­pyridinium hexa­fluorido­phosphate (2)**

This was prepared and crystallized in analogous manner to that for **1** using 2.508 g (1.019 mmol) of 3-cyano-1-methyl­pyridinium iodide and 1.873 g (1.223 mmol) of solid potassium hexa­fluorido­phosphate. M.p. 394 K.

**4-Cyano-1-methyl­pyridinium hexa­fluorido­phosphate (3)**

This was prepared and crystallized in analogous manner to that for **1** using 2.491 g (1.012 mmol) of 4-cyano-1-methyl­pyridinium iodide and 1.873 g (1.223 mmol) of solid potassium hexa­fluorido­phosphate. M.p. 418 K.

## Refinement details   

Crystal data, data collection and structure refinement details are summarized in Table 5 ▸. Crystals of **1** are twinned by a 180° rotation about the c* axis. Trial refinements of this structure with the single-component reflection file extracted from the twinned data set with *TWINABS* (Sheldrick, 2009 ▸) and the full 2-component reflection file showed the former to be more satisfactory. The anion in **2** is rotationally disordered by 38.2 (1)° about the F1—P1—F4 axis in an 0.848 (3):0.152 (3) ratio. The two components of the disorder were refined with restraints that their geometries be comparable. H atoms were placed in calculated positions and refined using a riding model: C—H = 0.98 Å with *U*_iso_(H) = 1.5*U*_eq_(C) for methyl H atoms, C—H = 0.95 Å with *U*_iso_(H) = 1.2*U*_eq_(C) for all other H atoms.

## Supplementary Material

Crystal structure: contains datablock(s) New_Global_Publ_Block, 1, 2, 3. DOI: 10.1107/S2056989018011003/hb7755sup1.cif

Structure factors: contains datablock(s) 1. DOI: 10.1107/S2056989018011003/hb77551sup2.hkl

Supporting information file. DOI: 10.1107/S2056989018011003/hb77551sup5.cml

Structure factors: contains datablock(s) 2. DOI: 10.1107/S2056989018011003/hb77552sup3.hkl

Supporting information file. DOI: 10.1107/S2056989018011003/hb77552sup6.cml

Structure factors: contains datablock(s) 3. DOI: 10.1107/S2056989018011003/hb77553sup4.hkl

Supporting information file. DOI: 10.1107/S2056989018011003/hb77553sup7.cml

CCDC references: 1859710, 1859709, 1859708

Additional supporting information:  crystallographic information; 3D view; checkCIF report

## Figures and Tables

**Figure 2:**
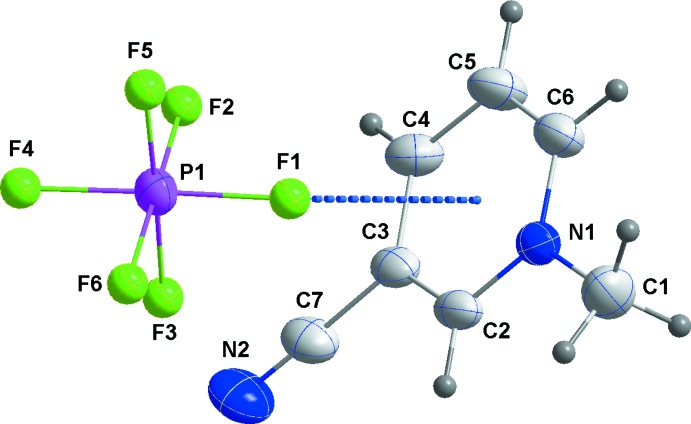
Perspective view of **2** with labeling scheme and 50% probability ellipsoids. Only the major orientation of the disordered anion is shown. The cation–anion inter­action is indicated by a dashed line.

**Figure 3:**
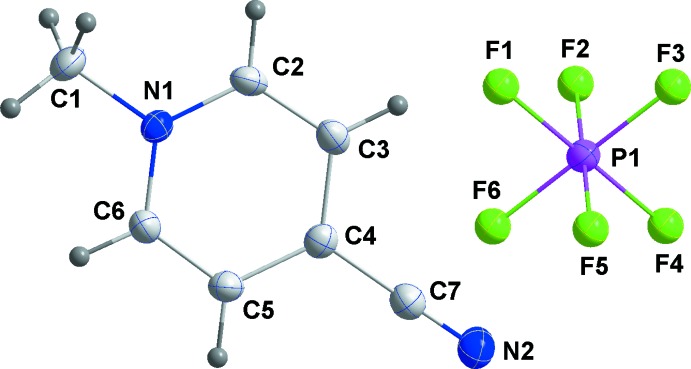
Perspective view of **3** with labeling scheme and 50% probability ellipsoids.

**Figure 9:**
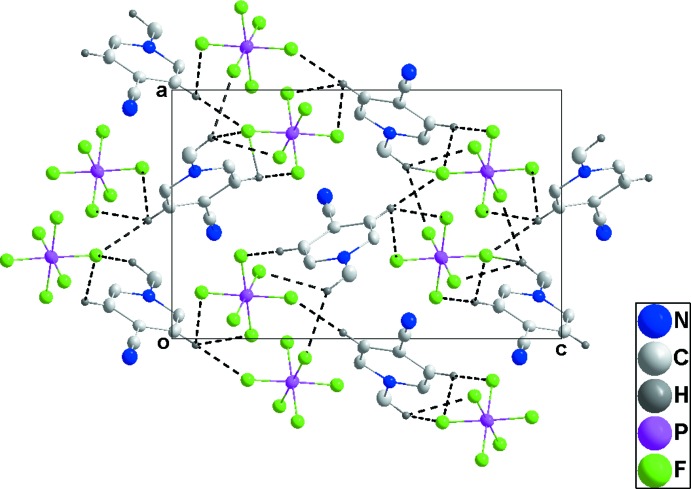
Packing of **3** viewed along the *b*-axis direction. C—H⋯F hydrogen bonds are shown by black dashed lines.

**Table 1:** Hydrogen-bond geometry (Å, °) for **1** [Chem scheme1]

*D*—H⋯*A*	*D*—H	H⋯*A*	*D*⋯*A*	*D*—H⋯*A*
C1—H1*A*⋯F4^i^	0.98	2.40	3.161 (3)	134
C1—H1*B*⋯F6^ii^	0.98	2.40	3.307 (3)	154
C4—H4⋯F6^iii^	0.95	2.41	3.319 (3)	160
C5—H5⋯F5^iv^	0.95	2.51	3.409 (3)	158

Symmetry codes: (i) 
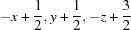
; (ii) 
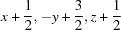
; (iii) 
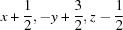
; (iv) 
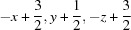
.

**Table 2:** Hydrogen-bond geometry (Å, °) for **2** [Chem scheme1]

*D*—H⋯*A*	*D*—H	H⋯*A*	*D*⋯*A*	*D*—H⋯*A*
C1—H1*B*⋯F4^i^	0.98	2.28	3.225 (5)	161
C2—H2⋯F6^i^	0.95	2.34	3.253 (4)	160
C6—H6⋯F6^ii^	0.95	2.53	3.389 (5)	150

Symmetry codes: (i) 
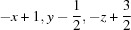
; (ii) 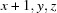.

**Table 3:** Hydrogen-bond geometry (Å, °) for **3** [Chem scheme1]

*D*—H⋯*A*	*D*—H	H⋯*A*	*D*⋯*A*	*D*—H⋯*A*
C5—H5⋯F5^i^	0.95	2.37	3.247 (2)	153
C3—H3⋯F3^ii^	0.95	2.46	3.106 (2)	126
C1—H1*C*⋯F1^iii^	0.98	2.51	3.208 (3)	128

Symmetry codes: (i) 
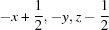
; (ii) 
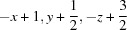
; (iii) 
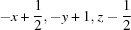
.

**Table 4:** Cation–anion inter­action energies (kcal mol^−1^)

Compound **1**		Compound **2**		Compound **3**	
*D*—H⋯*A*	Δ*E*_int_	*D*—H⋯*A*	Δ*E*_int_	*D*—H⋯*A*	Δ*E*_int_
C1—H1*A*⋯F4^i^	−19.0	C1—H1*B*⋯F4^iv^	−16.6	C5—H5⋯F5^vi^	−14.2
C1—H1*B*⋯F6^ii^	−15.9	C2—H2⋯F6^iv^	−16.6	C3—H3⋯F3^vii^	−15.3
C4—H4⋯F6	−15.7	C6—H6⋯F6^v^	−17.8	C1—H1*C*⋯F1^viii^	−16.7
C5—H5⋯F5^iii^	−15.9				

Symmetry codes: (i) −*x* + 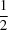, *y* + 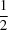, −*z* + 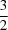; (ii) *x* + 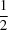, −*y* + 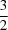, *z* + 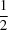; (iii) −*x* + 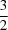, *y* + 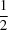, −*z* + 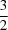; (iv) −*x* + 1, *y* − 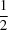, −*z* + 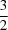; (v) *x* + 1, *y*, *z*; (vi) −*x* + 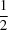, −*y*, *z* − 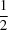; (vii) −*x* + 1, *y* + 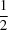, −*z* + 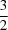; (viii) −*x* + 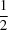, −*y* + 1, *z* − 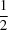.

**Table 5:** Experimental details

	**1**	**2**	**3**
Crystal data			
Chemical formula	C_7_H_7_N_2_^+^·PF_6_^−^	C_7_H_7_N_2_^+^·PF_6_^−^	C_7_H_7_N_2_^+^·PF_6_^−^
*M*_r_	264.12	264.12	264.12
Crystal system, space group	Monoclinic, *P*2_1_/*n*	Orthorhombic, *P*2_1_2_1_2_1_	Orthorhombic, *P*2_1_2_1_2_1_
Temperature (K)	150	150	150
*a*, *b*, *c* (Å)	6.5296 (5), 15.7145 (13), 9.5550 (7)	7.8484 (2), 10.8964 (2), 11.8669 (3)	8.5293 (6), 8.6264 (7), 13.3589 (10)
α, β, γ (°)	90, 93.327 (4), 90	90, 90, 90	90, 90, 90
*V* (Å^3^)	978.78 (13)	1014.85 (4)	982.91 (13)
*Z*	4	4	4
Radiation type	Cu *K*α	Cu *K*α	Mo *K*α
μ (mm^−1^)	3.21	3.09	0.34
Crystal size (mm)	0.20 × 0.17 × 0.06	0.26 × 0.19 × 0.15	0.26 × 0.19 × 0.13

Data collection			
Diffractometer	Bruker D8 VENTURE PHOTON 100 CMOS	Bruker D8 VENTURE PHOTON 100 CMOS	Bruker *SMART* *APEX* CCD
Absorption correction	Multi-scan (*TWINABS*; Sheldrick, 2009 ▸)	Multi-scan (*SADABS*; Bruker, 2015 ▸)	Multi-scan (*SADABS*; Bruker, 2015 ▸)
*T*_min_, *T*_max_	0.57, 0.84	0.59, 0.65	0.89, 0.96
No. of measured, independent and observed [*I* > 2σ(*I*)] reflections	12567, 1895, 1692	15204, 2009, 1970	19081, 2642, 2420
*R*_int_	0.040	0.034	0.033
(sin θ/λ)_max_ (Å^−1^)	0.618	0.618	0.686

Refinement			
*R*[*F* ^2^ > 2σ(*F* ^2^)], *wR*(*F* ^2^), *S*	0.042, 0.115, 1.07	0.036, 0.095, 1.08	0.031, 0.084, 1.13
No. of reflections	1895	2009	2642
No. of parameters	147	160	146
No. of restraints	0	8	0
H-atom treatment	H-atom parameters constrained	H-atom parameters constrained	H-atom parameters constrained
Δρ_max_, Δρ_min_ (e Å^−3^)	0.31, −0.33	0.35, −0.36	0.31, −0.20
Absolute structure	–	Flack *x* determined using 800 quotients [(*I* ^+^)−(*I* ^−^)]/[(*I* ^+^)+(*I* ^−^)] (Parsons *et al.*, 2013 ▸)	Flack *x* determined using 988 quotients [(*I* ^+^)−(*I* ^−^)]/[(*I* ^+^)+(*I* ^−^)] (Parsons *et al.*, 2013 ▸)
Absolute structure parameter	–	0.040 (6)	−0.01 (3)

Computer programs: *APEX2* and *SAINT* (Bruker, 2015 ▸), *CELL_NOW* (Sheldrick, 2008*b* ▸), *SHELXT* (Sheldrick, 2015*a* ▸), *SHELXL* (Sheldrick, 2015*b* ▸), *DIAMOND* (Brandenburg & Putz, 2012 ▸) and *SHELXTL* (Sheldrick, 2008*a* ▸).

## supplementary crystallographic information

### 2-Cyano-1-methylpyridinium hexafluoridophosphate (1) Crystal data

**Table:**

C_7_H_7_N_2_^+^·PF_6_^−^	*F*(000) = 528
*M**_r_* = 264.12	*D*_x_ = 1.792 Mg m^−^^3^
Monoclinic, *P*2_1_/*n*	Cu *K*α radiation, λ = 1.54178 Å
*a* = 6.5296 (5) Å	Cell parameters from 2191 reflections
*b* = 15.7145 (13) Å	θ = 7.3–71.9°
*c* = 9.5550 (7) Å	µ = 3.21 mm^−^^1^
β = 93.327 (4)°	*T* = 150 K
*V* = 978.78 (13) Å^3^	Plate, colourless
*Z* = 4	0.20 × 0.17 × 0.06 mm

### 2-Cyano-1-methylpyridinium hexafluoridophosphate (1) Data collection

**Table:**

Bruker D8 VENTURE PHOTON 100 CMOS diffractometer	1895 independent reflections
Radiation source: INCOATEC IµS micro–focus source	1692 reflections with *I* > 2σ(*I*)
Mirror monochromator	*R*_int_ = 0.040
Detector resolution: 10.4167 pixels mm^-1^	θ_max_ = 72.4°, θ_min_ = 5.4°
ω scans	*h* = −7→7
Absorption correction: multi-scan (*TWINABS*; Sheldrick, 2009)	*k* = −17→17
*T*_min_ = 0.57, *T*_max_ = 0.84	*l* = −10→8
12567 measured reflections	

### 2-Cyano-1-methylpyridinium hexafluoridophosphate (1) Refinement

**Table:**

Refinement on *F*^2^	Secondary atom site location: difference Fourier map
Least-squares matrix: full	Hydrogen site location: inferred from neighbouring sites
*R*[*F*^2^ > 2σ(*F*^2^)] = 0.042	H-atom parameters constrained
*wR*(*F*^2^) = 0.115	*w* = 1/[σ^2^(*F*_o_^2^) + (0.0632*P*)^2^ + 0.6053*P*] where *P* = (*F*_o_^2^ + 2*F*_c_^2^)/3
*S* = 1.07	(Δ/σ)_max_ < 0.001
1895 reflections	Δρ_max_ = 0.31 e Å^−^^3^
147 parameters	Δρ_min_ = −0.33 e Å^−^^3^
0 restraints	Extinction correction: SHELXL2014/7 (Sheldrick, 2015b), Fc^*^=kFc[1+0.001xFc^2^λ^3^/sin(2θ)]^-1/4^
Primary atom site location: structure-invariant direct methods	Extinction coefficient: 0.0045 (7)

### 2-Cyano-1-methylpyridinium hexafluoridophosphate (1) Special details

**Table:**

Experimental. Analysis of 2191 reflections having I/σ(I) > 13 and chosen from the full data set with *CELL_NOW* (Sheldrick, 2008) showed the crystal to belong to the monoclinic system and to be twinned by a 180° rotation about the *c** axis. The raw data were processed using the multi-component version of *SAINT* under control of the two-component orientation file generated by *CELL_NOW*.
Geometry. All esds (except the esd in the dihedral angle between two l.s. planes) are estimated using the full covariance matrix. The cell esds are taken into account individually in the estimation of esds in distances, angles and torsion angles; correlations between esds in cell parameters are only used when they are defined by crystal symmetry. An approximate (isotropic) treatment of cell esds is used for estimating esds involving l.s. planes.
Refinement. Refinement of F^2^ against ALL reflections. The weighted R-factor wR and goodness of fit S are based on F^2^, conventional R-factors R are based on F, with F set to zero for negative F^2^. The threshold expression of F^2^ > 2sigma(F^2^) is used only for calculating R-factors(gt) etc. and is not relevant to the choice of reflections for refinement. R-factors based on F^2^ are statistically about twice as large as those based on F, and R- factors based on ALL data will be even larger. H-atoms were placed in calculated positions (C—H = 0.95 - 0.98 Å) and included as riding contributions with isotropic displacement parameters 1.2 - 1.5 times those of the attached carbon atoms. Trial refinements with both the single-component data extracted with *TWINABS* and the full twinned data indicated that the former produced a more satisfactory model.

### 2-Cyano-1-methylpyridinium hexafluoridophosphate (1) Fractional atomic coordinates and isotropic or equivalent isotropic displacement parameters (Å^2^)

**Table:**

	*x*	*y*	*z*	*U*_iso_*/*U*_eq_	
N1	0.4176 (3)	0.86681 (11)	0.80398 (18)	0.0250 (4)	
N2	−0.0598 (3)	0.77157 (13)	0.7824 (2)	0.0388 (5)	
C1	0.3803 (4)	0.85654 (16)	0.9546 (2)	0.0349 (5)	
H1A	0.2619	0.8911	0.9776	0.052*	
H1B	0.5018	0.8752	1.0115	0.052*	
H1C	0.3527	0.7966	0.9743	0.052*	
C2	0.2757 (3)	0.83897 (13)	0.7044 (2)	0.0256 (4)	
C3	0.3078 (4)	0.84670 (14)	0.5644 (2)	0.0308 (5)	
H3	0.2084	0.8265	0.4957	0.037*	
C4	0.4873 (4)	0.88441 (14)	0.5246 (2)	0.0341 (5)	
H4	0.5129	0.8899	0.4282	0.041*	
C5	0.6281 (4)	0.91379 (14)	0.6259 (3)	0.0350 (5)	
H5	0.7506	0.9407	0.6000	0.042*	
C6	0.5899 (3)	0.90390 (14)	0.7655 (2)	0.0311 (5)	
H6	0.6876	0.9238	0.8355	0.037*	
C7	0.0906 (3)	0.80090 (14)	0.7509 (2)	0.0286 (5)	
P1	0.42105 (8)	0.58120 (3)	0.73007 (5)	0.0260 (2)	
F1	0.3952 (3)	0.66725 (10)	0.81616 (18)	0.0499 (4)	
F2	0.6643 (2)	0.59231 (10)	0.74158 (17)	0.0427 (4)	
F3	0.4065 (2)	0.63247 (11)	0.58661 (16)	0.0499 (4)	
F4	0.4482 (3)	0.49411 (10)	0.64785 (17)	0.0506 (4)	
F5	0.4308 (2)	0.52981 (10)	0.87364 (15)	0.0471 (4)	
F6	0.1770 (2)	0.56909 (11)	0.71904 (16)	0.0443 (4)	

### 2-Cyano-1-methylpyridinium hexafluoridophosphate (1) Atomic displacement parameters (Å^2^)

**Table:**

	*U*^11^	*U*^22^	*U*^33^	*U*^12^	*U*^13^	*U*^23^
N1	0.0252 (9)	0.0227 (9)	0.0271 (9)	0.0018 (7)	0.0011 (7)	−0.0016 (6)
N2	0.0349 (12)	0.0336 (11)	0.0478 (12)	−0.0043 (8)	0.0029 (9)	0.0027 (9)
C1	0.0381 (13)	0.0414 (13)	0.0248 (11)	0.0031 (10)	−0.0003 (9)	−0.0011 (9)
C2	0.0264 (11)	0.0195 (9)	0.0307 (10)	0.0033 (8)	−0.0006 (8)	0.0001 (8)
C3	0.0362 (12)	0.0249 (11)	0.0310 (11)	0.0002 (9)	−0.0023 (9)	0.0001 (8)
C4	0.0433 (14)	0.0273 (11)	0.0324 (12)	0.0021 (9)	0.0082 (10)	0.0018 (9)
C5	0.0323 (12)	0.0281 (11)	0.0453 (14)	−0.0012 (9)	0.0093 (10)	0.0001 (9)
C6	0.0273 (11)	0.0267 (11)	0.0391 (13)	−0.0001 (8)	0.0003 (9)	−0.0040 (9)
C7	0.0301 (12)	0.0250 (11)	0.0301 (10)	0.0003 (8)	−0.0033 (8)	0.0009 (8)
P1	0.0283 (3)	0.0249 (3)	0.0248 (3)	0.00132 (19)	0.0022 (2)	0.00086 (19)
F1	0.0546 (10)	0.0339 (8)	0.0603 (10)	0.0116 (7)	−0.0027 (7)	−0.0167 (7)
F2	0.0283 (8)	0.0477 (9)	0.0520 (9)	0.0000 (6)	0.0022 (6)	0.0001 (7)
F3	0.0482 (9)	0.0618 (10)	0.0400 (9)	0.0034 (7)	0.0036 (7)	0.0229 (7)
F4	0.0600 (10)	0.0387 (8)	0.0539 (9)	−0.0024 (7)	0.0104 (8)	−0.0192 (7)
F5	0.0528 (10)	0.0545 (9)	0.0347 (8)	0.0112 (7)	0.0074 (6)	0.0171 (7)
F6	0.0287 (8)	0.0613 (10)	0.0428 (8)	−0.0047 (6)	0.0017 (6)	0.0025 (7)

### 2-Cyano-1-methylpyridinium hexafluoridophosphate (1) Geometric parameters (Å, º)

**Table:**

N1—C6	1.338 (3)	C4—C5	1.375 (4)
N1—C2	1.361 (3)	C4—H4	0.9500
N1—C1	1.482 (3)	C5—C6	1.380 (3)
N2—C7	1.141 (3)	C5—H5	0.9500
C1—H1A	0.9800	C6—H6	0.9500
C1—H1B	0.9800	P1—F3	1.5881 (14)
C1—H1C	0.9800	P1—F5	1.5899 (14)
C2—C3	1.371 (3)	P1—F4	1.5931 (15)
C2—C7	1.442 (3)	P1—F2	1.5953 (15)
C3—C4	1.386 (3)	P1—F1	1.5967 (15)
C3—H3	0.9500	P1—F6	1.6020 (15)
			
C6—N1—C2	119.82 (19)	C6—C5—H5	120.3
C6—N1—C1	120.14 (19)	N1—C6—C5	121.1 (2)
C2—N1—C1	120.04 (18)	N1—C6—H6	119.4
N1—C1—H1A	109.5	C5—C6—H6	119.4
N1—C1—H1B	109.5	N2—C7—C2	177.1 (2)
H1A—C1—H1B	109.5	F3—P1—F5	178.89 (9)
N1—C1—H1C	109.5	F3—P1—F4	90.74 (9)
H1A—C1—H1C	109.5	F5—P1—F4	89.38 (9)
H1B—C1—H1C	109.5	F3—P1—F2	90.76 (9)
N1—C2—C3	121.1 (2)	F5—P1—F2	90.35 (8)
N1—C2—C7	117.79 (19)	F4—P1—F2	89.37 (9)
C3—C2—C7	121.1 (2)	F3—P1—F1	90.70 (9)
C2—C3—C4	119.0 (2)	F5—P1—F1	89.19 (9)
C2—C3—H3	120.5	F4—P1—F1	178.54 (10)
C4—C3—H3	120.5	F2—P1—F1	90.37 (9)
C5—C4—C3	119.4 (2)	F3—P1—F6	89.67 (8)
C5—C4—H4	120.3	F5—P1—F6	89.23 (9)
C3—C4—H4	120.3	F4—P1—F6	90.24 (9)
C4—C5—C6	119.5 (2)	F2—P1—F6	179.43 (9)
C4—C5—H5	120.3	F1—P1—F6	90.00 (9)
			
C6—N1—C2—C3	1.3 (3)	C2—C3—C4—C5	−0.5 (3)
C1—N1—C2—C3	−179.1 (2)	C3—C4—C5—C6	1.1 (3)
C6—N1—C2—C7	−178.81 (19)	C2—N1—C6—C5	−0.7 (3)
C1—N1—C2—C7	0.8 (3)	C1—N1—C6—C5	179.7 (2)
N1—C2—C3—C4	−0.7 (3)	C4—C5—C6—N1	−0.5 (3)
C7—C2—C3—C4	179.4 (2)		

### 2-Cyano-1-methylpyridinium hexafluoridophosphate (1) Hydrogen-bond geometry (Å, º)

**Table:**

*D*—H···*A*	*D*—H	H···*A*	*D*···*A*	*D*—H···*A*
C1—H1*A*···F4^i^	0.98	2.40	3.161 (3)	134
C1—H1*B*···F6^ii^	0.98	2.40	3.307 (3)	154
C4—H4···F6^iii^	0.95	2.41	3.319 (3)	160
C5—H5···F5^iv^	0.95	2.51	3.409 (3)	158

Symmetry codes: (i) −*x*+1/2, *y*+1/2, −*z*+3/2; (ii) *x*+1/2, −*y*+3/2, *z*+1/2; (iii) *x*+1/2, −*y*+3/2, *z*−1/2; (iv) −*x*+3/2, *y*+1/2, −*z*+3/2.

### 3-Cyano-1-methylpyridinium hexafluoridophosphate (2) Crystal data

**Table:**

C_7_H_7_N_2_^+^·PF_6_^−^	*D*_x_ = 1.729 Mg m^−^^3^
*M**_r_* = 264.12	Cu *K*α radiation, λ = 1.54178 Å
Orthorhombic, *P*2_1_2_1_2_1_	Cell parameters from 9953 reflections
*a* = 7.8484 (2) Å	θ = 3.7–72.4°
*b* = 10.8964 (2) Å	µ = 3.09 mm^−^^1^
*c* = 11.8669 (3) Å	*T* = 150 K
*V* = 1014.85 (4) Å^3^	Block, colourless
*Z* = 4	0.26 × 0.19 × 0.15 mm
*F*(000) = 528	

### 3-Cyano-1-methylpyridinium hexafluoridophosphate (2) Data collection

**Table:**

Bruker D8 VENTURE PHOTON 100 CMOS diffractometer	2009 independent reflections
Radiation source: INCOATEC IµS micro–focus source	1970 reflections with *I* > 2σ(*I*)
Mirror monochromator	*R*_int_ = 0.034
Detector resolution: 10.4167 pixels mm^-1^	θ_max_ = 72.3°, θ_min_ = 5.5°
ω scans	*h* = −9→9
Absorption correction: multi-scan (*SADABS*; Bruker, 2015)	*k* = −13→13
*T*_min_ = 0.59, *T*_max_ = 0.65	*l* = −14→14
15204 measured reflections	

### 3-Cyano-1-methylpyridinium hexafluoridophosphate (2) Refinement

**Table:**

Refinement on *F*^2^	Hydrogen site location: inferred from neighbouring sites
Least-squares matrix: full	H-atom parameters constrained
*R*[*F*^2^ > 2σ(*F*^2^)] = 0.036	*w* = 1/[σ^2^(*F*_o_^2^) + (0.0513*P*)^2^ + 0.5414*P*] where *P* = (*F*_o_^2^ + 2*F*_c_^2^)/3
*wR*(*F*^2^) = 0.095	(Δ/σ)_max_ < 0.001
*S* = 1.08	Δρ_max_ = 0.35 e Å^−^^3^
2009 reflections	Δρ_min_ = −0.36 e Å^−^^3^
160 parameters	Extinction correction: *SHELXL* (Sheldrick, 2015b), Fc^*^=kFc[1+0.001xFc^2^λ^3^/sin(2θ)]^-1/4^
8 restraints	Extinction coefficient: 0.0095 (11)
Primary atom site location: structure-invariant direct methods	Absolute structure: Flack *x* determined using 800 quotients [(*I*^+^)-(*I*^-^)]/[(*I*^+^)+(*I*^-^)] (Parsons *et al.*, 2013)
Secondary atom site location: difference Fourier map	Absolute structure parameter: 0.040 (6)

### 3-Cyano-1-methylpyridinium hexafluoridophosphate (2) Special details

**Table:**

Geometry. All esds (except the esd in the dihedral angle between two l.s. planes) are estimated using the full covariance matrix. The cell esds are taken into account individually in the estimation of esds in distances, angles and torsion angles; correlations between esds in cell parameters are only used when they are defined by crystal symmetry. An approximate (isotropic) treatment of cell esds is used for estimating esds involving l.s. planes.
Refinement. Refinement of F^2^ against ALL reflections. The weighted R-factor wR and goodness of fit S are based on F^2^, conventional R-factors R are based on F, with F set to zero for negative F^2^. The threshold expression of F^2^ > 2sigma(F^2^) is used only for calculating R-factors(gt) etc. and is not relevant to the choice of reflections for refinement. R-factors based on F^2^ are statistically about twice as large as those based on F, and R- factors based on ALL data will be even larger. H-atoms were placed in calculated positions (C—H = 0.95 - 0.98 Å) and included as riding contributions with isotropic displacement parameters 1.2 - 1.5 times those of the attached carbon atoms. The anion is rotationally disordered over two resolved sites about the F1···F4 axis in a 85/15 ratio. The disorder was refined with restraints that the two components have the same geometry.

### 3-Cyano-1-methylpyridinium hexafluoridophosphate (2) Fractional atomic coordinates and isotropic or equivalent isotropic displacement parameters (Å^2^)

**Table:**

	*x*	*y*	*z*	*U*_iso_*/*U*_eq_	Occ. (<1)
N1	0.9719 (3)	0.3953 (2)	0.6993 (2)	0.0309 (5)	
N2	0.5379 (5)	0.3571 (3)	0.4213 (3)	0.0543 (8)	
C1	0.9963 (5)	0.3420 (3)	0.8133 (3)	0.0446 (8)	
H1A	1.0606	0.2652	0.8073	0.067*	
H1B	0.8849	0.3254	0.8475	0.067*	
H1C	1.0594	0.4001	0.8606	0.067*	
C2	0.8312 (4)	0.3669 (3)	0.6408 (3)	0.0316 (7)	
H2	0.7464	0.3157	0.6733	0.038*	
C3	0.8100 (4)	0.4122 (3)	0.5329 (3)	0.0318 (7)	
C4	0.9332 (4)	0.4882 (3)	0.4868 (3)	0.0376 (7)	
H4	0.9196	0.5206	0.4131	0.045*	
C5	1.0763 (5)	0.5159 (4)	0.5499 (3)	0.0429 (8)	
H5	1.1621	0.5680	0.5198	0.051*	
C6	1.0937 (4)	0.4676 (3)	0.6563 (3)	0.0370 (7)	
H6	1.1924	0.4856	0.6995	0.044*	
C7	0.6579 (5)	0.3806 (3)	0.4718 (3)	0.0396 (8)	
P1	0.53561 (10)	0.67446 (7)	0.67835 (6)	0.0304 (2)	
F1	0.6869 (4)	0.6015 (3)	0.7338 (2)	0.0688 (8)	
F4	0.3803 (3)	0.7456 (3)	0.6233 (3)	0.0786 (9)	
F2	0.6590 (4)	0.7059 (3)	0.5769 (2)	0.0553 (8)	0.848 (3)
F3	0.4755 (4)	0.5537 (3)	0.6115 (3)	0.0647 (9)	0.848 (3)
F5	0.5888 (5)	0.7938 (3)	0.7430 (4)	0.0835 (14)	0.848 (3)
F6	0.4049 (4)	0.6385 (2)	0.7769 (2)	0.0522 (8)	0.848 (3)
F2A	0.599 (2)	0.664 (2)	0.5531 (6)	0.0553 (8)	0.152 (3)
F3A	0.481 (2)	0.5344 (7)	0.6895 (17)	0.0647 (9)	0.152 (3)
F5A	0.606 (2)	0.8092 (8)	0.679 (2)	0.0835 (14)	0.152 (3)
F6A	0.4950 (19)	0.6976 (14)	0.8095 (6)	0.0522 (8)	0.152 (3)

### 3-Cyano-1-methylpyridinium hexafluoridophosphate (2) Atomic displacement parameters (Å^2^)

**Table:**

	*U*^11^	*U*^22^	*U*^33^	*U*^12^	*U*^13^	*U*^23^
N1	0.0269 (11)	0.0281 (12)	0.0378 (13)	0.0013 (11)	0.0057 (11)	0.0006 (10)
N2	0.0478 (18)	0.0555 (19)	0.0597 (19)	−0.0142 (16)	−0.0071 (18)	−0.0133 (16)
C1	0.0425 (19)	0.0486 (19)	0.0428 (17)	−0.0023 (15)	−0.0016 (15)	0.0112 (15)
C2	0.0278 (15)	0.0254 (13)	0.0416 (16)	−0.0037 (12)	0.0070 (12)	−0.0030 (11)
C3	0.0314 (15)	0.0234 (13)	0.0406 (16)	−0.0029 (12)	0.0030 (13)	−0.0071 (12)
C4	0.0423 (19)	0.0321 (15)	0.0385 (16)	−0.0098 (14)	0.0023 (14)	−0.0003 (13)
C5	0.0374 (18)	0.0435 (19)	0.0477 (19)	−0.0147 (15)	0.0036 (15)	0.0047 (15)
C6	0.0286 (15)	0.0370 (17)	0.0453 (18)	−0.0060 (13)	0.0026 (13)	0.0000 (14)
C7	0.0397 (17)	0.0359 (16)	0.0431 (17)	−0.0079 (14)	−0.0002 (15)	−0.0096 (15)
P1	0.0261 (4)	0.0322 (4)	0.0331 (4)	0.0031 (3)	0.0033 (3)	0.0003 (3)
F1	0.0616 (15)	0.095 (2)	0.0500 (13)	0.0445 (15)	−0.0073 (12)	0.0000 (13)
F4	0.0455 (14)	0.090 (2)	0.100 (2)	0.0266 (14)	−0.0090 (14)	0.0258 (19)
F2	0.0416 (16)	0.078 (2)	0.0465 (14)	−0.0058 (14)	0.0129 (13)	0.0148 (14)
F3	0.0441 (13)	0.0667 (17)	0.083 (2)	−0.0172 (14)	0.0128 (17)	−0.0386 (17)
F5	0.0592 (17)	0.0662 (19)	0.125 (4)	−0.0103 (14)	0.011 (2)	−0.064 (2)
F6	0.0469 (15)	0.0530 (16)	0.0567 (15)	0.0087 (11)	0.0262 (13)	0.0062 (12)
F2A	0.0416 (16)	0.078 (2)	0.0465 (14)	−0.0058 (14)	0.0129 (13)	0.0148 (14)
F3A	0.0441 (13)	0.0667 (17)	0.083 (2)	−0.0172 (14)	0.0128 (17)	−0.0386 (17)
F5A	0.0592 (17)	0.0662 (19)	0.125 (4)	−0.0103 (14)	0.011 (2)	−0.064 (2)
F6A	0.0469 (15)	0.0530 (16)	0.0567 (15)	0.0087 (11)	0.0262 (13)	0.0062 (12)

### 3-Cyano-1-methylpyridinium hexafluoridophosphate (2) Geometric parameters (Å, º)

**Table:**

N1—C6	1.340 (4)	C5—C6	1.375 (5)
N1—C2	1.341 (4)	C5—H5	0.9500
N1—C1	1.485 (4)	C6—H6	0.9500
N2—C7	1.145 (5)	P1—F5	1.567 (3)
C1—H1A	0.9800	P1—F5A	1.569 (6)
C1—H1B	0.9800	P1—F2A	1.572 (6)
C1—H1C	0.9800	P1—F1	1.573 (2)
C2—C3	1.382 (5)	P1—F2	1.582 (2)
C2—H2	0.9500	P1—F4	1.586 (3)
C3—C4	1.385 (4)	P1—F3A	1.590 (6)
C3—C7	1.438 (5)	P1—F6	1.604 (2)
C4—C5	1.383 (5)	P1—F3	1.607 (3)
C4—H4	0.9500	P1—F6A	1.608 (6)
			
C6—N1—C2	121.7 (3)	F5A—P1—F1	101.8 (8)
C6—N1—C1	119.1 (3)	F2A—P1—F1	96.9 (7)
C2—N1—C1	119.2 (3)	F5—P1—F2	91.7 (2)
N1—C1—H1A	109.5	F1—P1—F2	88.03 (15)
N1—C1—H1B	109.5	F5—P1—F4	90.1 (2)
H1A—C1—H1B	109.5	F5A—P1—F4	79.4 (8)
N1—C1—H1C	109.5	F2A—P1—F4	83.6 (7)
H1A—C1—H1C	109.5	F1—P1—F4	178.71 (18)
H1B—C1—H1C	109.5	F2—P1—F4	92.93 (16)
N1—C2—C3	119.8 (3)	F5A—P1—F3A	172.8 (10)
N1—C2—H2	120.1	F2A—P1—F3A	95.4 (10)
C3—C2—H2	120.1	F1—P1—F3A	71.5 (7)
C2—C3—C4	119.7 (3)	F4—P1—F3A	107.3 (7)
C2—C3—C7	118.8 (3)	F5—P1—F6	90.9 (2)
C4—C3—C7	121.6 (3)	F1—P1—F6	93.12 (15)
C5—C4—C3	119.0 (3)	F2—P1—F6	177.11 (18)
C5—C4—H4	120.5	F4—P1—F6	85.88 (16)
C3—C4—H4	120.5	F5—P1—F3	178.3 (2)
C6—C5—C4	119.5 (3)	F1—P1—F3	90.83 (19)
C6—C5—H5	120.2	F2—P1—F3	88.94 (18)
C4—C5—H5	120.2	F4—P1—F3	88.3 (2)
N1—C6—C5	120.3 (3)	F6—P1—F3	88.39 (17)
N1—C6—H6	119.8	F5A—P1—F6A	85.2 (11)
C5—C6—H6	119.8	F2A—P1—F6A	171.6 (9)
N2—C7—C3	178.5 (4)	F1—P1—F6A	79.9 (5)
F5A—P1—F2A	87.9 (12)	F4—P1—F6A	99.8 (5)
F5—P1—F1	90.8 (2)	F3A—P1—F6A	91.0 (9)
			
C6—N1—C2—C3	0.3 (4)	C7—C3—C4—C5	179.6 (3)
C1—N1—C2—C3	−177.6 (3)	C3—C4—C5—C6	0.2 (5)
N1—C2—C3—C4	−0.9 (4)	C2—N1—C6—C5	0.5 (5)
N1—C2—C3—C7	−179.9 (3)	C1—N1—C6—C5	178.5 (3)
C2—C3—C4—C5	0.7 (5)	C4—C5—C6—N1	−0.8 (6)

### 3-Cyano-1-methylpyridinium hexafluoridophosphate (2) Hydrogen-bond geometry (Å, º)

**Table:**

*D*—H···*A*	*D*—H	H···*A*	*D*···*A*	*D*—H···*A*
C1—H1*B*···F4^i^	0.98	2.28	3.225 (5)	161
C2—H2···F6^i^	0.95	2.34	3.253 (4)	160
C6—H6···F6^ii^	0.95	2.53	3.389 (5)	150

Symmetry codes: (i) −*x*+1, *y*−1/2, −*z*+3/2; (ii) *x*+1, *y*, *z*.

### 4-Cyano-1-methylpyridinium hexafluoridophosphate (3) Crystal data

**Table:**

C_7_H_7_N_2_^+^·PF_6_^−^	*D*_x_ = 1.785 Mg m^−^^3^
*M**_r_* = 264.12	Mo *K*α radiation, λ = 0.71073 Å
Orthorhombic, *P*2_1_2_1_2_1_	Cell parameters from 9502 reflections
*a* = 8.5293 (6) Å	θ = 2.8–29.1°
*b* = 8.6264 (7) Å	µ = 0.34 mm^−^^1^
*c* = 13.3589 (10) Å	*T* = 150 K
*V* = 982.91 (13) Å^3^	Block, colourless
*Z* = 4	0.26 × 0.19 × 0.13 mm
*F*(000) = 528	

### 4-Cyano-1-methylpyridinium hexafluoridophosphate (3) Data collection

**Table:**

Bruker SMART APEX CCD diffractometer	2642 independent reflections
Radiation source: fine-focus sealed tube	2420 reflections with *I* > 2σ(*I*)
Graphite monochromator	*R*_int_ = 0.033
Detector resolution: 8.3333 pixels mm^-1^	θ_max_ = 29.2°, θ_min_ = 2.8°
φ and ω scans	*h* = −11→11
Absorption correction: multi-scan (*SADABS*; Bruker, 2015)	*k* = −11→11
*T*_min_ = 0.89, *T*_max_ = 0.96	*l* = −18→18
19081 measured reflections	

### 4-Cyano-1-methylpyridinium hexafluoridophosphate (3) Refinement

**Table:**

Refinement on *F*^2^	Secondary atom site location: difference Fourier map
Least-squares matrix: full	Hydrogen site location: inferred from neighbouring sites
*R*[*F*^2^ > 2σ(*F*^2^)] = 0.031	H-atom parameters constrained
*wR*(*F*^2^) = 0.084	*w* = 1/[σ^2^(*F*_o_^2^) + (0.0536*P*)^2^ + 0.0393*P*] where *P* = (*F*_o_^2^ + 2*F*_c_^2^)/3
*S* = 1.13	(Δ/σ)_max_ = 0.006
2642 reflections	Δρ_max_ = 0.31 e Å^−^^3^
146 parameters	Δρ_min_ = −0.20 e Å^−^^3^
0 restraints	Absolute structure: Flack *x* determined using 988 quotients [(*I*^+^)-(*I*^-^)]/[(*I*^+^)+(*I*^-^)] (Parsons *et al.*, 2013)
Primary atom site location: structure-invariant direct methods	Absolute structure parameter: −0.01 (3)

### 4-Cyano-1-methylpyridinium hexafluoridophosphate (3) Special details

**Table:**

Experimental. The diffraction data were obtained from 3 sets of 400 frames, each of width 0.5° in ω, colllected at φ = 0.00, 90.00 and 180.00° and 2 sets of 800 frames, each of width 0.45° in φ, collected at ω = –30.00 and 210.00°. The scan time was 15 sec/frame.
Geometry. All esds (except the esd in the dihedral angle between two l.s. planes) are estimated using the full covariance matrix. The cell esds are taken into account individually in the estimation of esds in distances, angles and torsion angles; correlations between esds in cell parameters are only used when they are defined by crystal symmetry. An approximate (isotropic) treatment of cell esds is used for estimating esds involving l.s. planes.
Refinement. Refinement of F^2^ against ALL reflections. The weighted R-factor wR and goodness of fit S are based on F^2^, conventional R-factors R are based on F, with F set to zero for negative F^2^. The threshold expression of F^2^ > 2sigma(F^2^) is used only for calculating R-factors(gt) etc. and is not relevant to the choice of reflections for refinement. R-factors based on F^2^ are statistically about twice as large as those based on F, and R- factors based on ALL data will be even larger. H-atoms attached to carbon were placed in calculated positions (C—H = 0.95 - 0.98 Å). All were included as riding contributions with isotropic displacement parameters 1.2 - 1.5 times those of the attached atoms.

### 4-Cyano-1-methylpyridinium hexafluoridophosphate (3) Fractional atomic coordinates and isotropic or equivalent isotropic displacement parameters (Å^2^)

**Table:**

	*x*	*y*	*z*	*U*_iso_*/*U*_eq_	
N1	0.3277 (2)	0.58167 (17)	0.44131 (12)	0.0197 (3)	
N2	0.5725 (2)	0.0199 (2)	0.39214 (14)	0.0317 (4)	
C1	0.2618 (3)	0.7396 (2)	0.45537 (17)	0.0280 (5)	
H1A	0.3475	0.8152	0.4583	0.042*	
H1B	0.2020	0.7431	0.5179	0.042*	
H1C	0.1925	0.7647	0.3991	0.042*	
C2	0.4065 (2)	0.5148 (3)	0.51769 (14)	0.0226 (4)	
H2	0.4191	0.5693	0.5790	0.027*	
C3	0.4688 (2)	0.3685 (2)	0.50746 (15)	0.0231 (4)	
H3	0.5244	0.3211	0.5609	0.028*	
C4	0.4481 (2)	0.2915 (2)	0.41667 (15)	0.0204 (4)	
C5	0.3665 (2)	0.3617 (2)	0.33903 (15)	0.0230 (4)	
H5	0.3519	0.3096	0.2771	0.028*	
C6	0.3075 (2)	0.5081 (2)	0.35368 (14)	0.0222 (4)	
H6	0.2517	0.5579	0.3012	0.027*	
C7	0.5161 (3)	0.1389 (2)	0.40273 (16)	0.0246 (4)	
P1	0.32732 (6)	0.01238 (6)	0.69107 (4)	0.02273 (14)	
F1	0.22985 (17)	0.16304 (16)	0.72363 (11)	0.0386 (4)	
F2	0.48612 (19)	0.1071 (2)	0.70429 (16)	0.0594 (5)	
F3	0.3350 (2)	−0.04134 (17)	0.80554 (10)	0.0471 (4)	
F4	0.42360 (18)	−0.13840 (19)	0.65850 (12)	0.0446 (4)	
F5	0.16725 (16)	−0.08286 (15)	0.67718 (10)	0.0326 (3)	
F6	0.31409 (19)	0.06562 (16)	0.57645 (10)	0.0403 (4)	

### 4-Cyano-1-methylpyridinium hexafluoridophosphate (3) Atomic displacement parameters (Å^2^)

**Table:**

	*U*^11^	*U*^22^	*U*^33^	*U*^12^	*U*^13^	*U*^23^
N1	0.0209 (7)	0.0168 (7)	0.0213 (8)	−0.0018 (7)	0.0018 (6)	0.0005 (6)
N2	0.0355 (10)	0.0287 (9)	0.0310 (10)	0.0060 (8)	−0.0003 (8)	0.0012 (8)
C1	0.0374 (12)	0.0178 (9)	0.0289 (12)	0.0031 (8)	0.0009 (9)	−0.0037 (8)
C2	0.0259 (9)	0.0248 (9)	0.0171 (8)	−0.0056 (8)	−0.0023 (7)	−0.0018 (8)
C3	0.0238 (9)	0.0243 (10)	0.0213 (10)	−0.0036 (8)	−0.0035 (7)	0.0035 (8)
C4	0.0186 (8)	0.0195 (8)	0.0229 (9)	−0.0027 (7)	0.0013 (7)	0.0010 (7)
C5	0.0280 (10)	0.0218 (9)	0.0192 (9)	−0.0021 (7)	−0.0015 (7)	−0.0023 (7)
C6	0.0255 (9)	0.0215 (8)	0.0195 (9)	−0.0004 (8)	−0.0031 (7)	0.0018 (7)
C7	0.0257 (10)	0.0261 (10)	0.0220 (10)	0.0001 (8)	−0.0008 (8)	0.0017 (8)
P1	0.0237 (2)	0.0204 (2)	0.0241 (3)	0.00204 (19)	−0.00338 (19)	−0.00227 (19)
F1	0.0487 (8)	0.0299 (7)	0.0372 (8)	0.0158 (6)	−0.0114 (7)	−0.0105 (6)
F2	0.0318 (7)	0.0491 (9)	0.0972 (15)	−0.0113 (7)	−0.0132 (10)	−0.0124 (10)
F3	0.0709 (10)	0.0456 (8)	0.0246 (7)	0.0168 (8)	−0.0175 (7)	0.0008 (6)
F4	0.0438 (8)	0.0377 (8)	0.0524 (10)	0.0207 (7)	−0.0057 (7)	−0.0131 (7)
F5	0.0298 (6)	0.0325 (6)	0.0357 (7)	−0.0074 (6)	−0.0027 (6)	0.0051 (6)
F6	0.0550 (10)	0.0379 (7)	0.0281 (7)	−0.0040 (7)	0.0091 (7)	0.0076 (6)

### 4-Cyano-1-methylpyridinium hexafluoridophosphate (3) Geometric parameters (Å, º)

**Table:**

N1—C6	1.343 (2)	C4—C5	1.388 (3)
N1—C2	1.351 (2)	C4—C7	1.451 (3)
N1—C1	1.486 (2)	C5—C6	1.374 (3)
N2—C7	1.142 (3)	C5—H5	0.9500
C1—H1A	0.9800	C6—H6	0.9500
C1—H1B	0.9800	P1—F2	1.5918 (16)
C1—H1C	0.9800	P1—F4	1.5985 (14)
C2—C3	1.376 (3)	P1—F3	1.5992 (15)
C2—H2	0.9500	P1—F6	1.6026 (15)
C3—C4	1.394 (3)	P1—F1	1.6030 (14)
C3—H3	0.9500	P1—F5	1.6042 (14)
			
C6—N1—C2	121.37 (17)	C4—C5—H5	120.7
C6—N1—C1	119.67 (17)	N1—C6—C5	120.78 (18)
C2—N1—C1	118.95 (17)	N1—C6—H6	119.6
N1—C1—H1A	109.5	C5—C6—H6	119.6
N1—C1—H1B	109.5	N2—C7—C4	178.6 (2)
H1A—C1—H1B	109.5	F2—P1—F4	90.63 (9)
N1—C1—H1C	109.5	F2—P1—F3	90.45 (10)
H1A—C1—H1C	109.5	F4—P1—F3	90.20 (8)
H1B—C1—H1C	109.5	F2—P1—F6	91.08 (10)
N1—C2—C3	120.56 (19)	F4—P1—F6	90.54 (9)
N1—C2—H2	119.7	F3—P1—F6	178.30 (10)
C3—C2—H2	119.7	F2—P1—F1	89.70 (9)
C2—C3—C4	118.32 (19)	F4—P1—F1	179.67 (9)
C2—C3—H3	120.8	F3—P1—F1	89.81 (9)
C4—C3—H3	120.8	F6—P1—F1	89.45 (8)
C5—C4—C3	120.40 (19)	F2—P1—F5	179.72 (10)
C5—C4—C7	120.02 (19)	F4—P1—F5	89.37 (9)
C3—C4—C7	119.56 (19)	F3—P1—F5	89.83 (8)
C6—C5—C4	118.57 (19)	F6—P1—F5	88.64 (8)
C6—C5—H5	120.7	F1—P1—F5	90.30 (8)
			
C6—N1—C2—C3	0.1 (3)	C3—C4—C5—C6	0.2 (3)
C1—N1—C2—C3	179.82 (18)	C7—C4—C5—C6	−178.13 (17)
N1—C2—C3—C4	−0.1 (3)	C2—N1—C6—C5	0.1 (3)
C2—C3—C4—C5	0.0 (3)	C1—N1—C6—C5	−179.63 (18)
C2—C3—C4—C7	178.32 (18)	C4—C5—C6—N1	−0.2 (3)

### 4-Cyano-1-methylpyridinium hexafluoridophosphate (3) Hydrogen-bond geometry (Å, º)

**Table:**

*D*—H···*A*	*D*—H	H···*A*	*D*···*A*	*D*—H···*A*
C5—H5···F5^i^	0.95	2.37	3.247 (2)	153
C3—H3···F3^ii^	0.95	2.46	3.106 (2)	126
C1—H1*C*···F1^iii^	0.98	2.51	3.208 (3)	128

Symmetry codes: (i) −*x*+1/2, −*y*, *z*−1/2; (ii) −*x*+1, *y*+1/2, −*z*+3/2; (iii) −*x*+1/2, −*y*+1, *z*−1/2.

## References

[bb41] Bockman, T. M. & Kochi, J. K. (1989). *J. Am. Chem. Soc.* **111**, 4669–4683.

[bb42] Bockman, T. M. & Kochi, J. K. (1992). *New J. Chem.* **16**, 39–49.

[bb1] Brandenburg, K. & Putz, H. (2012). *DIAMOND*, Crystal Impact GbR, Bonn, Germany.

[bb2] Bruker (2015). *APEX2* and *SAINT*. Bruker AXS Inc., Madison, Wisconsin, USA.

[bb3] Chai, J.-D. & Head-Gordon, M. (2008). *Phys. Chem. Chem. Phys.* **10**, 6615–6620. 10.1039/b810189b 18989472

[bb4] Chan, H., Chen, Y., Dai, M., Lu, C.-N., Wang, H.-F., Ren, Z.-G., Huang, Z.-J., Ni, C.-Y. & Lang, J.-P. (2012). *ChemEngComm.* **14**, 466–473.

[bb5] Glavcheva, Z., Umezawa, H., Okada, S. & Nakanishi, H. (2004). *Mater. Lett.* **58**, 2466–2471.

[bb6] Grimme, S. (2006). *J. Comput. Chem.* **27**, 1787–1799. 10.1002/jcc.20495 16955487

[bb7] Groom, C. R., Bruno, I. J., Lightfoot, M. P. & Ward, S. C. (2016). *Acta Cryst.* B**72**, 171–179. 10.1107/S2052520616003954 PMC4822653 27048719

[bb39] Hardacre, C., Holbrey, J. D., Mullan, C. L., Nieuwenhuyzen, M., Reichert, W. M., Seddon, K. R. & Teat, S. J. (2008). *New J. Chem.* **32**, 1953–1967.

[bb40] Hardacre, C., Holbrey, J. D., Mullan, C. L., Nieuwenhuyzen, M., Youngs, T. G. A., Bowron, D. T. & Teat, S. J. (2010). *Phys. Chem. Chem. Phys.* **12**, 1842–1853. 10.1039/b921160h 20145851

[bb8] Johnson, E. R., Keinan, S., Mori-Sánchez, P., Contreras-García, J., Cohen, A. J. & Yang, W. (2010). *J. Am. Chem. Soc.* **132**, 6498–6506. 10.1021/ja100936w PMC2864795 20394428

[bb9] Jurečka, P., Černý, J., Hobza, P. & Salahub, D. (2007). *J. Comput. Chem.* **28**, 555–569. 10.1002/jcc.20570 17186489

[bb10] Kammer, M. N., Koplitz, L. V. & Mague, J. T. (2012*a*). *Acta Cryst.* E**68**, o2514. 10.1107/S1600536812032230 PMC3414965 22904952

[bb11] Kammer, M. N., Koplitz, L. V. & Mague, J. T. (2013). *Acta Cryst.* E**69**, o1281. 10.1107/S1600536813019302 PMC3793776 24109363

[bb12] Kammer, M. N., Mague, J. T. & Koplitz, L. V. (2012*b*). *Acta Cryst.* E**68**, o2409. 10.1107/S1600536812030449 PMC3414330 22904863

[bb13] Koplitz, L. V., Bay, K. D., DiGiovanni, N. & Mague, J. T. (2003). *J. Chem. Cryst.* **33**, 391–402.

[bb14] Koplitz, L. V., Mague, J. T., Kammer, M. N., McCormick, C. A., Renfro, H. E. & Vumbaco, D. J. (2012). *Acta Cryst.* E**68**, o1653. 10.1107/S1600536812019460 PMC3379252 22719450

[bb15] Mague, J. T., Ivie, R. M., Hartsock, R. W., Koplitz, L. V. & Spulak, M. (2005). *Acta Cryst.* E**61**, o851–o853.

[bb16] Marenich, A. V., Cramer, C. J. & Truhlar, D. G. (2009). *J. Phys. Chem. B*, **113**, 4538–4543. 10.1021/jp809094y 19253989

[bb17] McCormick, C. A., Nguyen, V. D., Koplitz, L. V. & Mague, J. T. (2014). *Acta Cryst.* E**70**, o811. 10.1107/S1600536814014421 PMC4120581 25161591

[bb18] McCormick, C. A., Nguyen, V. D., Renfro, H. E., Koplitz, L. V. & Mague, J. T. (2013). *Acta Cryst.* E**69**, o981–o982. 10.1107/S1600536813014025 PMC3685115 23795134

[bb19] Nguyen, V. D., McCormick, C. A., Koplitz, L. V. & Mague, J. T. (2014). *Acta Cryst.* E**70**, o756–o757. 10.1107/S1600536814012860 PMC4120629 25161550

[bb20] Nguyen, V. D., McCormick, C. A., Mague, J. T. & Koplitz, L. V. (2015*a*). *Acta Cryst.* E**71**, o852–o853. 10.1107/S2056989015019155 PMC4645073 26594561

[bb21] Nguyen, V. D., McCormick, C. A., Pascal, R. A., Mague, J. T. & Koplitz, L. V. (2015b). *Acta Cryst.* E**71**, o854–o855. 10.1107/S2056989015019167 PMC4645077 26594562

[bb22] Nguyen, V. D., McCormick, C. A., Vaccaro, F. A., Riley, K. E., Stephenson, C. J., Mague, J. T. & Koplitz, L. V. (2016). *Polyhedron*, **114**, 428–434.

[bb23] Parsons, S., Flack, H. D. & Wagner, T. (2013). *Acta Cryst.* B**69**, 249–259. 10.1107/S2052519213010014 PMC3661305 23719469

[bb24] Riley, K. E., Pitoňák, M., Jurečka, P. & Hobza, P. (2010). *Chem. Rev.* **110**, 5023–5063. 10.1021/cr1000173 20486691

[bb25] Riley, K. E., Tran, K. A., Lane, P., Murray, J. S. & Politzer, P. (2016). *J. Comput. Sci.* **17**, 273–284.

[bb26] Riley, K. E., Vondrášek, J. & Hobza, P. (2007). *Phys. Chem. Chem. Phys.* **9**, 5555–5560. 10.1039/b708089a 17957311

[bb27] Schröder, H., Hühnert, J. & Schwabe, T. (2017). *J. Chem. Phys.* **146**, 044115. 10.1063/1.4974840 28147528

[bb28] Sedlak, R., Janowski, T., Pitoňák, M., Řezáč, J., Pulay, P. & Hobza, P. (2013). *J. Chem. Theory Comput.* **9**, 3364–3374. 10.1021/ct400036b PMC3789125 24098094

[bb29] Sheldrick, G. M. (2008*a*). *Acta Cryst.* A**64**, 112–122. 10.1107/S0108767307043930 18156677

[bb30] Sheldrick, G. M. (2008*b*). *CELL_NOW*, University of Göttingen, Göttingen, Germany.

[bb31] Sheldrick, G. M. (2009). *TWINABS*, University of Göttingen, Göttingen, Germany.

[bb32] Sheldrick, G. M. (2015*a*). *Acta Cryst.* A**71**, 3–8.

[bb33] Sheldrick, G. M. (2015*b*). *Acta Cryst.* C**71**, 3–8.

[bb34] Shen, J., Zhang, C., Yu, T., An, L. & Fu, Y. (2014). *Cryst. Growth Des.* **14**, 6337–6342.

[bb35] Vaccaro, F. A., Koplitz, L. V. & Mague, J. T. (2015). *Acta Cryst.* E**71**, o697–o698. 10.1107/S2056989015016011 PMC4647419 26594429

[bb36] Wang, N., Wang, J.-G., Min, A.-J. & Fu, Y.-W. (2012). *Acta Cryst.* E**68**, m164. 10.1107/S1600536812001377 PMC3274894 22346841

[bb43] Yu, T.-L., An, L., Zhang, L., Shen, J.-J., Fu, Y.-B. & Fu, Y.-L. (2014). *Cryst. Growth Des.* **14**, 3875–3879.

[bb37] Zhu, D. & Kochi, J. K. (1999). *Organometallics*, **18**, 161–172.

